# Building National Capacity for Dementia Caregiving Research: The NIA Edward R. Roybal Centers

**DOI:** 10.1093/geroni/igaa057.1888

**Published:** 2020-12-16

**Authors:** Karina Davidson, Susan Hughes, Jeffrey Kaye, Scott Halpern, Kenneth Hepburn, Kathi Heffner

**Affiliations:** 1 Northwell Health, New York, New York, United States; 2 University of Illinois at Chicago, Chicago, Illinois, United States; 3 Layton Alzheimer’s Disease Center, Portland, Oregon, United States; 4 University of Pennsylvania, Philadelphia, Pennsylvania, United States; 5 Emory University, Atlanta, Georgia, United States; 6 University of Rochester Medical Center, Rochester, New York, United States

## Abstract

The NIA Edward R. Roybal Centers for Translational Research in the Behavioral and Social Sciences of Aging aim to translate and integrate basic behavioral and social research findings into principle-driven interventions aimed at innovatively improving both the lives of older people and the capacity of institutions to adapt to societal aging. Newly funded Centers focus on interventions to promote caregiving mastery, integrate the use of technology in care support to improve assessments and interventions in care provision, develop behavioral interventions to reduce isolation and promote social connectedness in caregivers, promote health in racial/ethnic minorities, and apply insights from data science and behavioral economics to improve palliative care delivery and long-term support facilities for persons with dementia and their caregivers. Center leaders will present an overview of their cutting-edge, early-stage research projects based on the NIH Stage model conceptual framework and discuss implications for improving care of caregivers and patients.

